# Parsing the Regulatory Network between Small RNAs and Target Genes in Ethylene Pathway in Tomato

**DOI:** 10.3389/fpls.2017.00527

**Published:** 2017-04-11

**Authors:** Yunxiang Wang, Qing Wang, Lipu Gao, Benzhong Zhu, Zheng Ju, Yunbo Luo, Jinhua Zuo

**Affiliations:** ^1^Key Laboratory of the Vegetable Postharvest Treatment of Ministry of Agriculture, Beijing Vegetable Research Center, Beijing Academy of Agriculture and Forestry SciencesBeijing, China; ^2^Beijing Key Laboratory of Fruits and Vegetable Storage and Processing, Beijing Vegetable Research Center, Beijing Academy of Agriculture and Forestry SciencesBeijing, China; ^3^Key Laboratory of Biology and Genetic Improvement of Horticultural Crops (North China) of Ministry of Agriculture, Beijing Vegetable Research Center, Beijing Academy of Agriculture and Forestry SciencesBeijing, China; ^4^Key Laboratory of Urban Agriculture (North) of Ministry of Agriculture, Beijing Vegetable Research Center, Beijing Academy of Agriculture and Forestry SciencesBeijing, China; ^5^Laboratory of Postharvest Molecular Biology of Fruits and Vegetables, Department of Food Biotechnology, College of Food Science and Nutritional Engineering, China Agricultural UniversityBeijing, China

**Keywords:** ethylene, microRNAs, target, high-throughput sequencing, regulatory network

## Abstract

Small RNAs are a class of short non-coding endogenous RNAs that play essential roles in many biological processes. Recent studies have reported that microRNAs (miRNAs) are also involved in ethylene signaling in plants. *LeERF1* is one of the ethylene response factors (ERFs) in tomato that locates in the downstream of ethylene signal transduction pathway. To elucidate the intricate regulatory roles of small RNAs in ethylene signaling pathway in tomato, the deep sequencing and bioinformatics methods were combined to decipher the small RNAs landscape in wild and sense-/antisense-*LeERF1* transgenic tomato fruits. Except for the known miRNAs, 36 putative novel miRNAs, 6 trans-acting short interfering RNAs (ta-siRNAs), and 958 natural antisense small interfering RNAs (nat-siRNAs) were also found in our results, which enriched the tomato small RNAs repository. Among these small RNAs, 9 miRNAs, and 12 nat-siRNAs were differentially expressed between the wild and transgenic tomato fruits significantly. A large amount of target genes of the small RNAs were identified and some of them were involved in ethylene pathway, including AP2 TFs, auxin response factors, F-box proteins, ERF TFs, APETALA2-like protein, and MADS-box TFs. Degradome sequencing further confirmed the targets of miRNAs and six novel targets were also discovered. Furthermore, a regulatory model which reveals the regulation relationships between the small RNAs and their targets involved in ethylene signaling was set up. This work provides basic information for further investigation of the function of small RNAs in ethylene pathway and fruit ripening.

## Introduction

Small RNAs are a class of non-coding endogenous RNAs ranged from 20 to 24 nucleotides (nt) that play essential roles in plant growth and development, signal transduction, response to biotic and abiotic stresses and other biological processes (Rhoades et al., 2002; Jones-Rhoades et al., 2006; Tomato Genome Consortium, 2012). MicroRNAs (miRNAs) and small-interfering RNAs (siRNAs) are two mainly classes of small RNAs divided on the difference of their precursor structures and biosynthetic pathways (Carthew and Sontheimer, 2009). Mature miRNAs are evolved from miRNA genes with the action of Dicer-like 1 (DCL1), Hua Enhancer 1 (HEN1), and HASTY proteins (Jones-Rhoades et al., 2006; Xie et al., 2015). SiRNAs are derived from long double-stranded RNAs (dsRNAs) and could be classed to heterochromatic siRNAs (hc-siRNAs), trans-acting short interfering RNAs (ta-siRNAs) and natural antisense siRNAs (nat-siRNAs; Chen, 2009). Recent studies showed that small RNAs can negatively regulate gene expression at the post-transcriptional level based on two possible mechanisms: transcript cleavage and translational repression (Sunkar et al., 2007; Couzigou and Combier, 2016).

As a climacteric fruit model, tomato has been widely used to study the molecular mechanisms of fruit ripening and senescence as well as ethylene biosynthesis and signal transduction. Recently, increasing studies showed that small RNAs are also involved in regulating ethylene signal transduction (Pilcher et al., 2007; Moxon et al., 2008; Zhang et al., 2011; Zuo et al., 2012). For example, Moxon et al. (2008) found that one of the target genes of miR156 was CNR, which belongs to SBP-box family transcription factors (TFs), and the target gene of miR172 was AP2. It has been reported that the expression of genes that encode miRNAs is regulated at the transcriptional level by various transcriptional factors (Yant et al., 2010; Baek et al., 2013). For example, EIN3, a key transcription factor in ethylene signaling, directly binds to the promoter region of miR164 and represses its transcription (Li et al., 2013).

ERFs were a class of TFs located in the downstream of ethylene signal transduction pathways that function in diverse plant growth and metabolism processes as well as in the biotic and abiotic stress response, such as ethylene (Wu et al., 2002; Pirrello et al., 2006), high salt (Park et al., 2001; Wang et al., 2004), drought and low temperature, and so on (Qin et al., 2004; Zhang et al., 2007). Given that the miRNAs were also involved in the ethylene signaling pathways, there may be some relationships between miRNAs and ERFs. The high-throughput sequencing technology has been widely used to explore the functions of miRNA and siRNAs due to its high throughputs and accuracy (An et al., 2011; Cao et al., 2014; Thiebaut et al., 2014). In this study, High-throughput sequencing of small RNAs and degradome sequencing were used to gain a better understanding of the relationship between ethylene and small RNAs using wild type and *LeERF1* transgenic tomato fruits. MiRNAs expression patterns were profiled and their targets were conferred; the regulatory network model between the small RNAs and ethylene was set up. This research provides more evidences for understanding the regulatory pathways of miRNAs in the network of fruit ripening.

## Materials and methods

### Sample collection and preparation

Wild type (*Solanum lycopersicum* cv. zhongshu4) and sense-/antisense-*LeERF1* transgenic tomato plants (Li et al., 2007) were grown in the greenhouse at standard conditions. The Fruits at breaker stage were used in the experiment (Supplementary Figure S1). Pooled mesocarp tissues from three groups were flash frozen in liquid nitrogen and stored at −80°C until further analysis.

### Small RNA (sRNA) quantification and qualification

The RNA samples were extracted using Trizol. Nanodrop, Qubit 2.0, and Agilent 2100 bioanalyzer were used to detect the purity, concentration and integrity of RNA samples, respectively, to ensure the use of qualified samples for sequencing. RNA purity was checked using the NanoPhotometer® spectrophotometer (IMPLEN, CA, USA). RNA concentration was measured using Qubit® RNA Assay Kit in Qubit®2.0 Flurometer (Life Technologies, CA, USA). RNA integrity was assessed using the RNA Nano 6000 Assay Kit of the Agilent Bioanalyzer 2100 system (Agilent Technologies, CA, USA).

### Library preparation for small RNA sequencing

A total amount of 1.5 μg RNA per sample was used as input material for the RNA sample preparations. Sequencing libraries were generated using NEBNext®Ultra™ small RNA Sample Library Prep Kit for Illumina®(NEB, USA) following manufacturer's recommendations and index codes were added to attribute sequences to each sample. Briefly, first of all, ligated the 3′ SR Adaptor, mixed 3′ SR Adaptor for Illumina, RNA and Nuclease-Free Water, mixture system after incubationa for 2 min at 70 degrees in a preheated thermal cycler, Tube was transferred to ice. Then, add 3′ Ligation Reaction Buffer (2X) and 3′ Ligation Enzyme Mix ligate the 3′ SR Adaptor, incubated for 1 h at 25°C in a thermal cycler. To prevent adaptor-dimer formation, the SR RT Primer hybridizes to the excess of 3′ SR Adaptor (that remains free after the 3′ ligation reaction) and transforms the single stranded DNA adaptor into a double-stranded DNA molecule. sRNAs (18–30 nucleotides in length) were separated from the total RNAs by polyacrylamide gel electrophoresis (PAGE). The small RNA molecules were then ligated with 5′ and 3′ adaptor and used for reverse transcription and subsequent PCR. The final PCR product was purified and sequenced by Illumina Cluster Station and Illumina Genome Analyzer (SanDiego, CA, USA).

### Clustering and sequencing

The clustering of the index-coded samples was performed on a cBot Cluster Generation System using TruSeq PE Cluster Kit v4-cBot-HS (Illumina) according to the manufacturer's instructions. After cluster generation, the library preparations were sequenced on an Illumina Hiseq 2500 platform and pair-end reads were generated. The sequencing results were deposited in the Sequence Read Archive (SRA) at the NCBI database (accession number: SRP094091).

### Quality control

The quality control of raw data (raw reads) in fastq format has been performed by using in-house scripts written in Perl. Reads containing adapter and poly-N sequences and reads with low quality from raw data were removed. Then reads were cleaned by removing the sequences smaller than 18 nt or longer than 30 nt. At the same time, Q20, Q30, GC-content, and sequence duplication level of the clean data were calculated. All the downstream analyses were based on clean data with high quality.

### Bioinformatic analysis of sequencing data

The Clean Reads were aligned with Silva database, GtRNAdb database, Rfam database, and Repbase database respectively to filter ribosomal RNA (rRNA), transfer RNA (tRNA), small nuclear RNA (snRNA), small nucleolar RNA (snoRNA), repeat sequences, and other ncRNA using Bowtie tools. The remaining reads were used to detect known miRNAs and new miRNAs predicted by comparing with known miRNAs from miRBase. RNAFold tools were used to predict the secondary structure of all new miRNAs.

### Identification of siRNA and putative novel miRNA

The adapter reads of the Solexa sequencing results were removed (Supplementary Figure S2). And reads larger than 30 nt and smaller than 18 nt were discarded. All high quality reads were considered as significant and further analyzed. Small RNA reads were mapped to tomato genome with mapping tool bowtie, all tomato genome annotation information is downloaded from ITAG2.3 include repeats and protein-coding regions (http://solgenomics.net/organism/Solanum_lycopersicum/genome). Six libraries are pooled together for miRNA prediction. The potential miRNA loci were analyzed using MIREAP software (version 0.2) with default parameters followed by additional manual check criteria that included: the miRNA sequence length should be between 18 and 26 nt; the maximal free energy allowed for a miRNA precursor (−18 kcal/mol); flank sequence length of miRNA precursor (100 nt); the predicted mature miRNA reads count should be large than 10 and reading counts ratio for miRNA^*^/miRNA should be small than 0.1. The unique reads left were aligned with known miRNAs from miRBase 21.0 (http://www.mirbase.org/). Phased small RNAs and nat-siRNAs were predicted as described in the previous studies (Chen et al., 2007; Zhou et al., 2009). All the reading counts were normalized to per million of total mapped reads (TPM).

### Target gene functional annotation

Gene function was annotated based on the following databases: Nr (NCBI non-redundant protein sequences); Nt (NCBI non-redundant nucleotide sequences); Pfam (Protein family); KOG/COG (Clusters of Orthologous Groups of proteins); Swiss-Prot (A manually annotated and reviewed protein sequence database); KO (KEGG Ortholog database); GO (GeneOntology).

### Quantification of small RNAs expression levels and differential expression analysis

Small RNA expression levels were estimated by TPM for each sample: sRNA were mapped back onto the reference genome, and read count for each small RNA was obtained from the mapping results. For the samples with biological replicates, differential expression analysis of two conditions/groups was performed using the DESeq R package (1.10.1). DESeq provide statistical routines for determining differential expression in digital miRNA expression data using a model based on the negative binomial distribution. The resulting *P*-values were adjusted using the Benjamini and Hochberg's approach for controlling the false discovery rate. MiRNA with an adjusted *p* < 0.05 and |log_2_(fold change)| ≥ 1 were assigned as differentially expressed (Anders and Huber, 2010).

### GO enrichment analysis

Gene Ontology (GO) enrichment analysis of the differentially expressed genes (DEGs) was implemented by the GOseq R packages based on Wallenius non-central hyper-geometric distribution (Young et al., 2010), which can adjust to gene length bias in DEGs.

### KEGG pathway enrichment analysis

KEGG (Kanehisa et al., 2008) is a database resource for understanding high-level functions and utilities of the biological system, such as the cell, the organism, and the ecosystem, from molecular-level information, especially large-scale molecular datasets generated by genome sequencing and other high-throughput experimental technologies (http://www.genome.jp/kegg/). We used KOBAS (Mao et al., 2005) software to test the statistical enrichment of differential expression genes in KEGG pathways.

## Results

### Overview of the small RNA libraries from tomato fruit

To identify small RNAs and analyze their functions in ethylene pathway, the deep sequencing technology with Illumina Hiseq 2500 platform (Biomarker Technologies, China) was performed in wild and sense-/antisense-*LeERF1* transgenic tomato fruits at breaker stage. A total of 19.31, 19.80, 17.28, 15.24, 14.61, 14.60 million raw reads in CK1 (wild 1), CK2 (wild 2), F1 (sense-*LeERF1* transgenic tomato 1), F2 (sense-*LeERF1* transgenic tomato 2), R1 (antisense-*LeERF1* transgenic tomato 1), and R2 (antisense-*LeERF1* transgenic tomato 2) were generated, respectively. After removing low quality and contaminated reads, poly A-containing sequences, sequences outside of 18–30 nt, 3′ and 5′ adaptors sequences, 15.75 (CK1), 16.88 (CK2), 13.79 (F1), 13.05 (F2), 12.63 (R1), 12.94 (R2) million clean reads were remained for further analysis. Then the small RNAs were categorized into miRNAs, ribosomal (r) RNAs, transfer (t) RNAs, small nuclear (sn) RNAs, small nucleolar (sno) RNAs, repeat regions, exon and intron RNA based on genomic location and function analysis (Table 1).

**Table 1:** **Small RNAs profiling and classification in six tomato fruit groups**.

**Types**	**CK1**	**CK2**	**F1**	**F2**	**R1**	**R2**
Total	15,753,031	16,876,616	13,793,259	13,050,621	12,633,453	12,938,234
miRNA	606,491	678,439	529,661	514,194	506,601	500,709
rRNA	363,895	334,157	348,969	268,843	294,359	318,280
tRNA	73,283	69,194	68,966	58,727	42,953	60,809
snRNA	4,726	5,906	5,517	5,220	4,802	5,752
snoRNA	7,876	8,100	8,275	7,264	5,938	6,951
Repeat	2,451,172	2,683,382	2,008,299	1,958,898	1,867,224	1,935,560
NAT	1,228,736	1,400,759	1,213,807	1,070,151	1,174,911	1,125,626
TAS	40,958	37,129	37,242	40,457	41,690	37,521
exon:+	677,380	573,805	537,937	587,278	517,972	491,653
exon:−	247,323	275,089	219,313	216,640	228,665	238,064
intron:+	849,088	916,400	760,009	726,920	720,107	720,660
intron:−	521,425	567,054	464,833	445,026	423,221	437,312
Other	8,680,677	9,327,202	7,590,433	7,151,003	6,805,010	7,059,338

**Figure 1:**
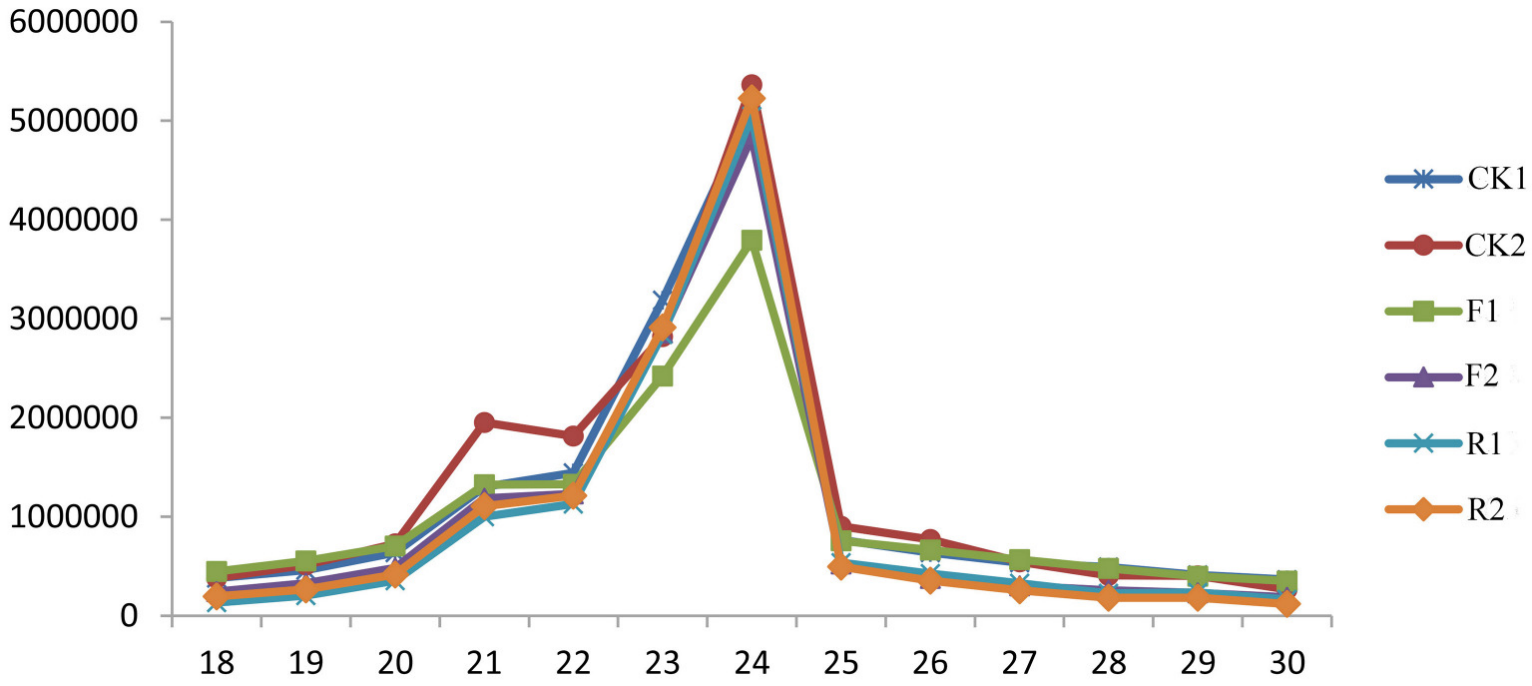
**Length distribution of small RNAs in wild (CK1, CK2), sense-*LeERF1* (F1, F2), and antisense-*LeERF1* (R1, R2) tomato fruit**.

The size distribution is one of the distinct features of the small RNAs libraries from different plants. In our experiments, the length of small RNAs ranges from 18 to 30 nt and the most abundant group of small RNAs have length 21–24 nt in all the six libraries (Figure 1). There is no obvious difference of the length distribution between wild and *LeERF1* transgenic tomato. Among the 21–24 nt size small RNAs, 24-nt size class has the highest abundance, accompanied with the 23-nt sRNAs as the second largest groups, which is in accordance with that of rice (Morin et al., 2008), Arabidopsis (Rajagopalan et al., 2006), and our previous results (Zuo et al., 2012, 2013).

### Identification of miRNAs and siRNAs in tomato

To identify miRNAs and siRNAs in tomato, the clean sequences were aligned with the tomato small RNAs database (http://ted.bti.cornell.edu/cgi-bin/TFGD/sRNA/home.cgi) and the latest miRNA database (http://www.mirbase.org/, Release 21). In total, 178 known miRNAs belonging to 108 families were obtained in our libraries. Among the 108 families, 46 miRNA families (Supplementary Table S1) were registered in miRBase as belonging to *S. lycopersicum* and other 62 families (Supplementary Table S2) were less conserved and first identified in tomato (Supplementary Figure S3). Most of the miRNA families belonging to *S. lycopersicum* in miRBase are composed of more than one member. For instance, miR156 and miR482 were the largest ones with seven members in the families in this study. MiR171 and miR319 were the second largest family with six members. On the other hand, except for the miR548 family, other less conserved miRNA families had only one member detected in this study. Sequence length statistical results showed that the 21-nt miRNAs were the main type of the 178 known miRNAs.

In addition, 36 putative novel miRNAs with hairpin structures renamed as miRZ101 to miRZ136 were predicted and all of them were found to have star sequences (Table 2 and Supplementary Table S3). The length of the putative novel miRNAs were 18–24 and 24 nt miRNAs accounted for the predominance. Most of the first nucleotide of the putative novel miRNAs were A, which was in accordance with previous study that 24 nt miRNAs used to had an A as the first nucleotide (Jain et al., 2014). The minimum folding free energies varied from −149.8 to −28.3 kcal/mol (Supplementary Table S3).

**Table 2:** **Putative novel miRNAs found in tomato**.

**MiRNA**	**Length**	**Sequence**	**Chromosome**	**Star**	**MEF (kcal/mol)**
miRZ101	22	uaacuucgucuagcucgccuuc	10	+	−70
miRZ102	24	guagagaacucuagaaccuucuag	10	+	−84.1
miRZ103	24	aaaggacuccuagauuucucuagu	11	+	−93.9
miRZ104	24	aaagacuguucaauuacugcuuga	11	+	−28.3
miRZ105	24	uauguccuuuaacuuugagugugc	12	+	−110.4
miRZ106	24	uuaguauaguauaagugugucucu	12	+	−57.1
miRZ107	24	acacacucugcauucaauuaaauu	12	+	−63.4
miRZ108	24	acguugcucagacucuucaaaaau	12	+	−60.3
miRZ109	22	auuuauggcuaugaauuugagu	12	+	−62.9
miRZ110	24	uuaguauaguauaagugugucucu	1	+	−41.7
miRZ111	24	uuaguuuaauuaagaugugucucu	1	+	−105.2
miRZ112	21	gcacggcagauaguuauuggc	1	+	−114.6
miRZ113	24	guagagaacucuagaaccuucuag	1	+	−71.4
miRZ114	24	aagcgaugacuuuagugaccuagu	1	+	−39.9
miRZ115	22	cacggucguaccuugacaaggc	2	+	−77.8
miRZ116	22	uuguuucuguuuuuguuugagu	2	+	−149.8
miRZ117	23	guugcucggacucuucaaaaaug	2	+	−69.1
miRZ118	20	auaacacaaaucugagccuc	2	+	−56.5
miRZ119	22	agugacucgcucgaucuuucuu	3	+	−64
miRZ120	24	uuucgucuugaaaguuugcccaug	4	+	−58.3
miRZ121	24	auuuccgaucaaacuuaaacuguu	4	+	−40.8
miRZ122	23	guugcucgaacucuucaaaaaug	5	+	−62.7
miRZ123	24	augugaucgcuguaaugaccuuac	5	+	−132.9
miRZ124	24	ucgagggucuaucagaaacaacau	6	+	−50.8
miRZ125	18	accugguugauccugcga	6	+	−73.3
miRZ126	24	guugcucgaacucuucaaaaaugu	6	+	−78.9
miRZ127	24	uuuucuaucggaacuaucaugugu	6	+	−69.3
miRZ128	21	ucaacgcugcacucaaucaug	7	+	−75.2
miRZ129	24	aagacguuugaaucugaaaaagau	8	+	−57.7
miRZ130	23	uuauacuauacuaagguccuauu	8	+	−117
miRZ131	24	cgagugcucauuccacagauaagu	8	+	−64.2
miRZ132	24	auacaucgguuacuugauagacgu	8	+	−109.4
miRZ133	24	uuaguauaguauaagugugucucu	8	+	−103.1
miRZ134	24	ugaaaucgagaugugauguagagg	9	+	−59.9
miRZ135	23	uucuucugacucauuuacuuuag	9	+	−54.2
miRZ136	24	augcucuagucuuugaacgacagg	9	+	−58.7

Moreover, several conserved and species-specific endogenous siRNAs were also characterized in our libraries. Ta-siRNAs are a special class of siRNAs that generated from TAS gene transcripts and mediated by miRNA (Xie et al., 2005; Yoshikawa et al., 2005; Li et al., 2012). On the basis of the conservation of the TAS genes in plants, three TAS5 gene family members: TAS5, TAS5b, and TAS5d (TAS5b and TAS5d were found in our previous study; Zuo et al., 2016), all miR482 targets, were identified (Table 3). Surprisingly, one more TAS5 family member (TAS5e) and two more TAS genes (TAS11a and TAS11b), triggered by sly-miR6024, are reported in our results (Table 4). In addition, 19 potential phased small RNAs and 958 nat-siRNAs were also found in this study (Supplementary Tables S4,S5).

**Table 3:** **The conserved TAS5 family in tomato fruit**.

**Name**	**Chromosome**	**Length**	**Start**	**End**	**Phased abundance**	**related miRNA**
sly-TAS5	6	539	423,570	424,108	3,954	sly-miR482d-3p
sly-TAS5b	2	644	21,186,658	21,187,301	530	sly-miR482d-3p
sly-TAS5d	8	917	58,262,775	58,263,691	4,729	sly-miR482e-3p

*Three TAS5 family members were found and were located in Chromosome 6, 2, and 8 separately. “Phased abundance” means the abundance of phased sequence and the “related miRNA” related the miRNAs that mediated TAS*.

**Table 4:** **The novel TAS families in tomato fruit**.

**Name**	**Chromosome**	**Start**	**End**	**Length**	**Phased abundance**	**related miRNA**
sly-TAS5e	11	48,467,984	48,468,816	833	22,190	sly-miR482b
sly-TAS11a	5	2,500,975	2,501,555	581	389	sly-miR6024
sly-TAS11b	11	51,986,458	51,986,681	224	137	sly-miR6024

*Three members belong to two TAS families (TAS5, TAS11) were found and located in Chromosome 11, 5, and 11 separately. “Phased abundance” means the abundance of phased sequence and the “related miRNA” related the miRNAs that mediated TAS*.

### The effect of overexpression sense-/antisense-*LeERF1* on small RNA profiles

To evaluate the regulatory roles of *LeERF1*on miRNA expression, differential expression of miRNAs among the wild and sense-/antisense-*LeERF1* transgenic tomato were analyzed. After normalization using a RPM method, the miR399a was found to have significant different accumulation between wild type and sense-*LeERF1* transgenic tomato fruits. The expression of the miR399a was down-regulated in sense-*LeERF1* transgenic fruit (**Figure 4A**). MiR8990 and the novel miRZ118 were the two miRNAs significant differently expressed between wild type and antisense-*LeERF1* transgenic tomato fruits, and their accumulations decreased in the transgenic fruit. Totally, there were nine miRNAs having significant differential expression between sense-*LeERF1* and antisense-*LeERF1* transgenic fruit. Among them, miR399a and miR8263-5p were up-regulated in antisense-*LeERF1* transgenic fruits. Meanwhile, other seven miRNAs including miR7484, miR319a, miR95-5p, miR8990, miR2569-5p, and two putative novel miRNAs (miRZ118 and miRZ131) were down-regulated.

Besides, 12 nat-siRNAs were found to show differential expression patterns. Compared with wild type fruits, most of the nat-siRNAs showed lower expression in sense-*LeERF1* tomato fruits, only two of them increased (**Figure 4B**). However, among the differentially expressed nat-siRNAs, more than half of them had higher expression levels in antisense-*LeERF1* transgenic fruits.

### Target gene identification of the miRNAs

**Figure 2:**
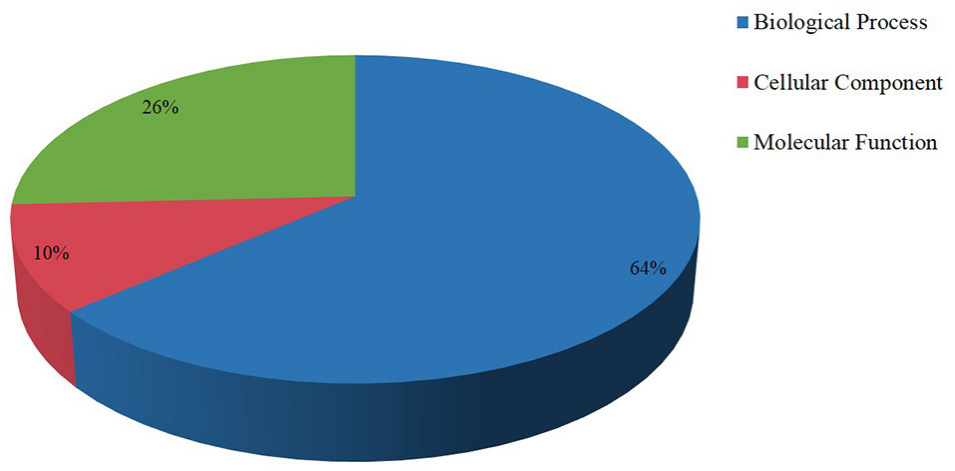
**Targets of miRNAs in tomato fruit**. Most targets of the miRNAs were involved biological process.

MiRNAs regulate gene expression mainly through cleaving mRNA or inhibiting the translation process of the targets gene, so identification and analysis of the target genes were the basis to study the function of miRNAs. Bioinformatics prediction and high-throughput degradome sequencing were the two main methods to find the targets gene. Using bioinformatic prediction method, a total of 103 target genes that involved in biological process, cellular component, and molecular function were found and most of them were identified to participate in biological process (Figure 2). Previous studies indicated that the targets of conserved miRNAs were also conservative and most of the miRNA families had not only one target site (Jin et al., 2008; Lu et al., 2008), which was also found in our study. For example, the targets of miR166a are homeobox-leucine zipper protein Revoluta, homeobox-leucine zipper protein ATHB-14 and pentatricopeptide repeat-containing protein At5g25630. Meanwhile, one target gene was often cleaved by two or more miRNAs. For instance, AP2 is the target of miR172 and miR8737, miR319 and miR159 share the same target GAMYB. Among the identified target genes, 16 targets were found to be involved in ethylene (Supplementary Table S6) and most of them were AP2 TFs. Another class of targets was auxin response factors including ARF10, ARF16, ARF17 and ARF18. Two F-box proteins (F-box protein 6, F-box protein At3g07870-like), two ethylene-responsive factors (RAP2-7-like) and an APETALA2-like protein were also predicted.

**Figure 3:**
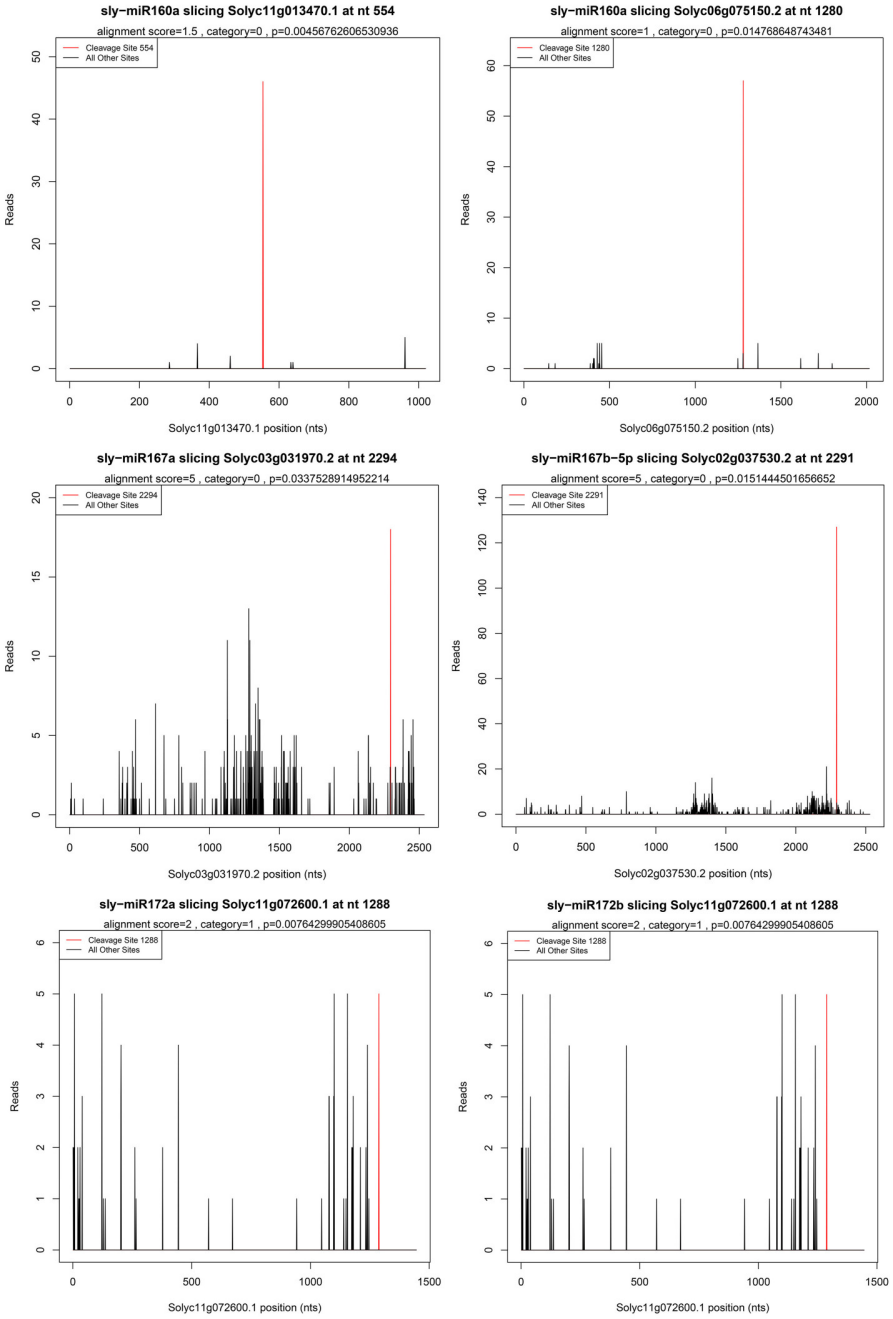
**Target plots of miRNA targets confirmed by degradome sequencing**. Six new genes were found to be targets of five miRNAs.

High-throughput degradome sequencing is a new technology to identify miRNAs targets and is successfully applied in Arabidopsis, rice (Addo-Quaye et al., 2008; Li et al., 2010). In this study, a total of 55 cleavage sites associated with 41 miRNAs were detected and seven target genes cleaved by five miRNAs were identified to be related to ethylene synthesis and signal transduction, including five auxin response factors (ARFs), one AP2 TF, and one ERF TF (Supplementary Table S7). Except for the known targets, six new targets were identified. The representative target plots of new targets were shown in Figure 3.

In addition, 389 genes were predicted to be the targets of nat-siRNAs, and 22 of them were found to participate in ethylene pathway (Supplementary Table S8). Ethylene-responsive TFs, MADS-box TFs, F-box proteins were the main targets involved in fruit ripening. Moreover, 55 targets of the ta-siRNAs were also predicted and two of them (Solyc02g092020.1 and Solyc02g082320.1) were related to ethylene pathway.

### Target parsing and small RNA regulatory network analysis in tomato

To investigate the network between the miRNAs and their targets, cytoscape platform was employed. In the network, it could be clearly seen that miR6024 had 11 targets and among the targets two of them were also the targets of miR482 (Figure 5). In addition, miR6024 and miR6027-3p shared one common targets. Moreover, miR6022 and miR528 shared most of their targets and they also had common targets with miR6023 and miR8527.

To comprehensively understand the functions of the miRNAs, ta-siRNAs, and nat-siRNAs involved in ethylene synthesis and signal transduction, all the predicted target genes of small RNAs were screened carefully and a regulatory model including small RNAs, the targets and their main functions was set up (**Figure 6**). As shown in **Figure 6**, it could clearly been seen that the AP2 TFs that involved in ethylene signaling were the targets of miR172a, miR172b, and miR8737. Ethylene-responsive transcription factors were the target of miR172a, miR172b and the nat-siRNAs renamed as nat-siRNA-G2009, nat-siRNA-G2010, nat-siRNA-G2011, nat-siRNA-G2012, nat-siRNA-G2013, and nat-siRNA-G2014. Meanwhile, the F-Box proteins and MADS-Box TFs that participated in ethylene signaling were the targets of miR394, miRZ131, nat-siRNA-G2015 to nat-siRNA-G2017, sly-TAS5d, phased small RNA001 and nat-siRNA-G2001 to nat-siRNA-G2005, respectively. Moreover, the auxin response factors that indirectly control ethylene signaling were the targets of miR160a and the nat-siRNA-G2006 and nat-siRNA-G2007.

## Discussion

Small RNAs are a class of non-coding RNAs that play vital roles in growth and development, signal transduction, biotic and abiotic stresses (Jones-Rhoades et al., 2006; Dalmay, 2010; Mohorianu et al., 2011; Zuo et al., 2012; Pashkovskiy and Ryazansky, 2013). Numerous studies have demonstrated that miRNAs were involved in the regulation of diverse physiological processes by repressing the expression of their target genes. Ethylene is an important endogenous hormone and plays important roles in fruit development and ripening. As a model plant, tomato has been widely used to study the molecular mechanisms of ethylene biosynthesis and signal transduction (Giovannoni, 2004; Osorio et al., 2011), and through the study on ripening-related mutants or transgenic plant, many advances have been achieved. ERFs were a class of TFs located in the downstream of ethylene signal transduction pathways, and as one of the members of ERF class, *LeERF1* had been showed to mediate fruit maturation and softening, enhance resistance to osmotic stress and improve plant tolerance to fungal invasion (Li et al., 2007; Lu et al., 2011; Pan et al., 2013). To better understand the relationship between *LeERF1* and small RNAs in ethylene pathway, high-throughput sequencing was employed in the sense-/antisense-*LeERF1* transgenic tomato fruits and many ethylene-related small RNAs as well as their target genes were found.

### High-throughput sequencing of tomato fruit

In the past decades, miRNAs identification and their biological roles analysis were the mainly focused research fields. In tomato, 46 miRNA families were identified and registered in the miRBase database (http://www.mirbase.org/). It is well-known that many small RNAs have temporal expression patterns (Chen, 2009; Rubio-Somoza et al., 2009) and many studies had not detected all the 46 miRNA families (Candar-Cakir et al., 2016; Wu et al., 2016). In this study, the 46 families were all identified though some miRNAs did not found in all libraries, such as miR169 that only detected in wild tomatoes. This result indicated that the high-throughput sequencing had superiority in the identification of small RNA. Meanwhile, we identified 62 less conservative miRNAs that had not previous been found in tomato but documented in the miRBase for other species. For instance, miR861 was found in Arabidopsis (Fahlgren et al., 2007) and miR8010 were registered for potato (Zhang et al., 2013). MiR440, miR528, miR2922 and miR1049, miR1222, miR1063 were detected in rice and moss, respectively (Liu et al., 2005; Sunkar et al., 2005; Talmor-Neiman et al., 2006; Axtell et al., 2007; Sanan-Mishra et al., 2009).

In addition, 41 putative novel miRNAs not identified in other reports were also predicted in this study. The hairpin structures were found and the minimal folding free energies (MFEs) were −149.8 to −28.3 kcal/mol, indicated that the hairpin structures were stable. MiRNAs with detected stars were more likely to predict to be bona fide novel miRNAs (Wu et al., 2016). The renamed putative novel miRNAs in our libraries all had stars, suggested the accuracy of the novel miRNAs. Most of the putative novel miRNAs were 24 nt in length. The 24 nt small RNAs were reported to mainly match to the promoter regions of ripening-associated genes (Tomato Genome Consortium, 2012) and its high percentage in the putative novel miRNAs may imply regulatory roles of the novel miRNAs in ethylene pathway.

Overall, the discovery of less conserved and putative novel miRNAs in tomato provided enriched insight into the plant miRNA dataset. In addition, one previously reported TAS5 gene (Li et al., 2012) and two other TAS5 genes (TAS5b, TAS5d, found in our other study) were detected in our libraries (Table 3). Moreover, another TAS5 (TAS5e) with the target sly-miR482b and two more TAS genes (Named TAS11a and TAS11b) triggered by sly-miR6024 were also found in our libraries (Table 4).

### Differential expression profiles of the small RNAs

**Figure 4:**
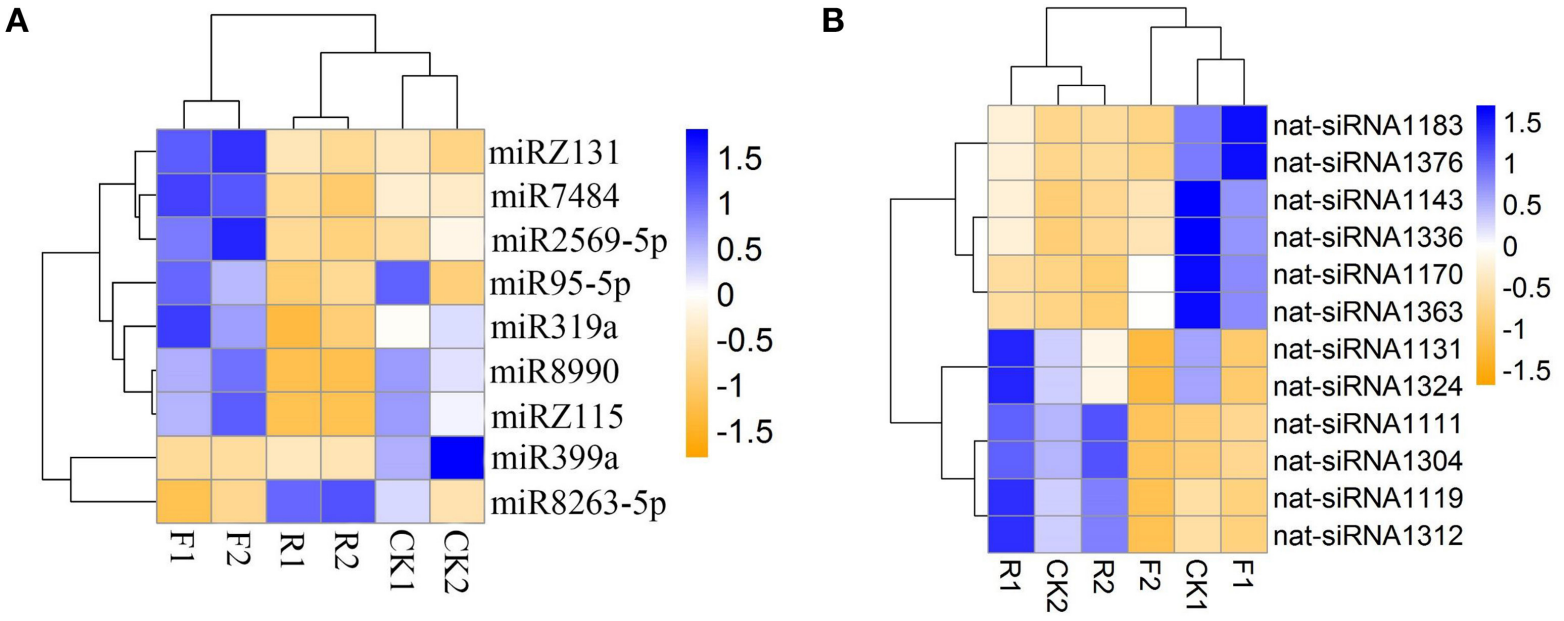
**Expression profiles of the significantly differentially expressed miRNAs (A)** and nat-siRNAs **(B)** in wild (CK1, CK2), overexpression sense-*LeERF1* (F1, F2), and antisense-*LeERF1* (R1, R2) tomato fruit.

It is well-known that many small RNAs have temporal expression patterns (Chen, 2009; Rubio-Somoza et al., 2009). Differential expression patterns of small RNAs can be regarded as an index for estimating the regulation contributions. We analyzed small RNAs expression in the wild and sense-/antisense-*LeERF1* transgenic tomato. It is worth to noting that 14 miRNAs had significant difference expression between the wild and transgenic tomato. Among them, four miRNA families had a significant different accumulation between wild and sense-*LeERF1* transgenic tomato fruits and 10 miRNAs differentially expressed in the response to antisense-*LeERF1*, indicating their specific roles in fruit ripening (Figure 4A).

**Figure 5:**
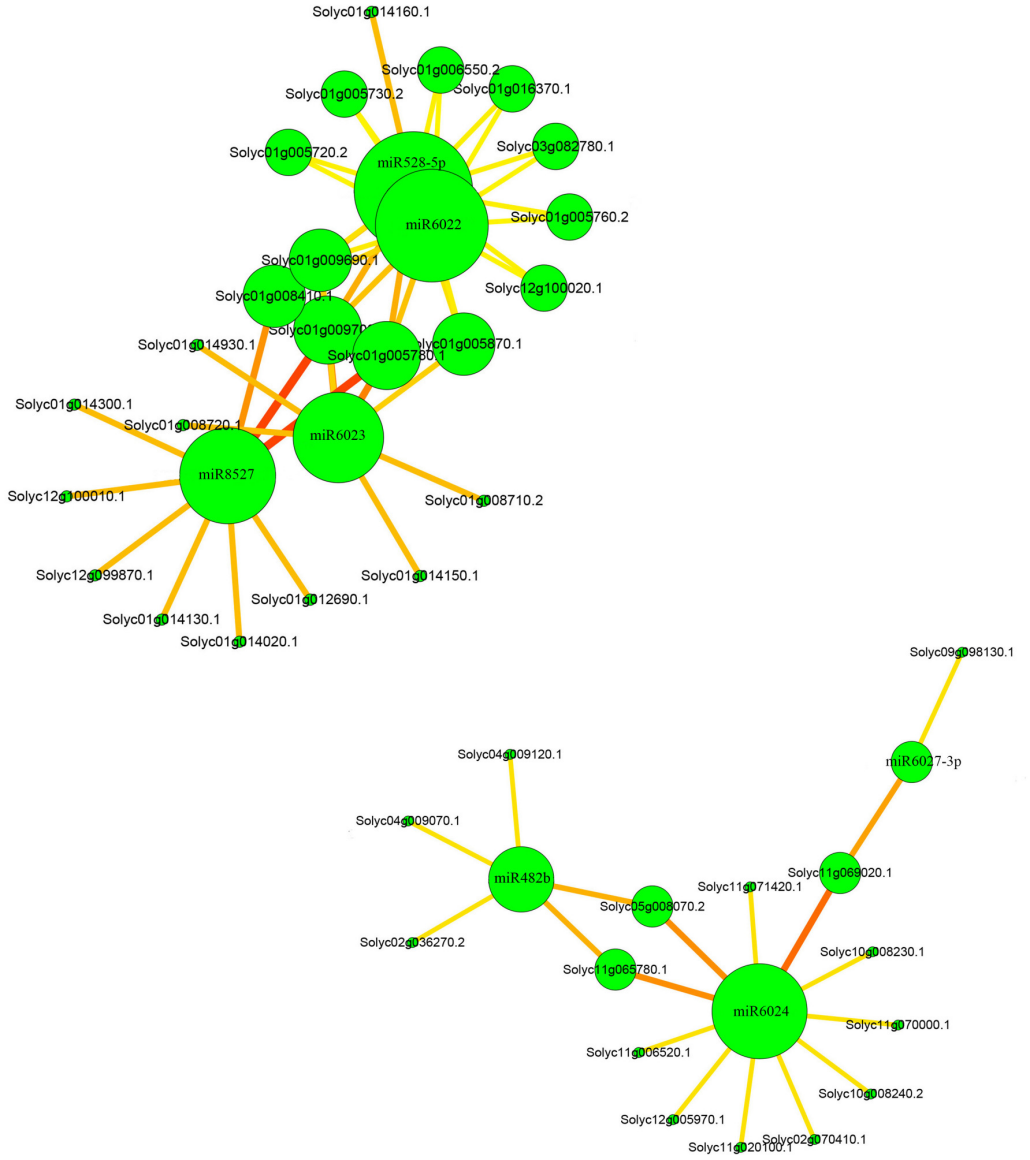
**Relationships between miRNAs and their targets**.

It has been reported that miR399 was involved in plant response to phosphate starvation (Fujii et al., 2005; Chiou et al., 2006) and its accumulation increased during fruit development in tomato (Gao et al., 2015). In this study, the miR399a is down-regulated in sense-*LeERF1* transgenic fruit, which indicated that miR399 may play an important role in ethylene signal transduction pathway. Totally, there were nine miRNAs having significant different expression between sense-*LeERF1* and antisense-*LeERF1* transgenic fruit. Among them, miR399a and miR8263-5p were up-regulated in antisense-*LeERF1* transgenic fruits, meanwhile, other nine miRNAs, including miR7484, miR319a, miR95-5p, miR8990, miR2569-5p, and two putative novel miRNAs (miRZ118 and miRZ131) were down-regulated. miR319 has been reported to control leaf development and morphogenesis through regulating transmission control protocol (TCP) transcription factors (Palatnik et al., 2003). In this study, the target of miR319 was predicted to be GAMYB, which was also related to ethylene pathway. According to our results, miR319 may also participate in ethylene signaling pathway.

Besides, 12 nat-siRNAs were found to show differential expression patterns. Compared with wild fruits, most of the nat-siRNAs showed lower expression levels in sense-*LeERF1* tomatoes, and only two of them increased. However, among the nat-siRNAs, more than half of them had higher expression levels in antisense-*LeERF1* transgenic fruits (Figure 4B).

### Small RNAs participated in ethylene pathway

To study the function of small RNAs in ethylene pathway, bioinformatic prediction, and degradome sequencing were also used in wild and sense-/antisense-*LeERF1* transgenic tomato. Results showed that most of the targets were identified to participate in various biological processes (Figure 2). AP2 transcription factors, AP2-like ethylene-responsive transcription factors, ethylene-responsive transcription factor were TFs belong to AP2/EREBP transcription factors family involved in ethylene signaling pathway and they were the main targets of miR172 family, which was also reported in Arabidopsis and tomato (Wu et al., 2009; Cheng et al., 2016). The AP2/EREBP transcription factors were also the target of miR5658 (Cheng et al., 2016). However, in this study, the miR5658 was not detected and miR8737 were predicted to target AP2/ERF TFs. F-Box proteins were reported to regulate ethylene signaling in Arabidopsis (Wang et al., 2009). It was also been reported that the F-Box proteins were the targets of miR393 in Arabidopsis (Liu et al., 2008). However, in this study, miR393 was not found and F-Box proteins were predicted to be the target of miR394. In addition, auxin response factors genes cleaved by miR160 were also found in our results. In Arabidopsis, ARF6 and ARF8, ARF16, and ARF17 were reported to be the targets of miR167 and miR160, respectively. In this study, ARF8 cleaved by miR167 was found via degradome sequencing. However, ARF6 was not identified for miR167, perhaps because the abundance of cleaved products was too low to be detected. Unexpectedly, ARF10 and ARF18 were identified to be the targets of miR160. Surprisingly, six new targets were found (Figure 3).

Compared with the miRNAs, most targets of the ta-siRNAs and nat-siRNAs in tomato were not verified yet. The distribution of the targets of ta-siRNAs and nat-siRNAs was different from that of the miRNAs, and a great part of the targets were predicted to be involve in all kinds of metabolic processes which were consistent with the previous studies (Zhai et al., 2011; Li et al., 2012; Shivaprasad et al., 2012; Zuo et al., 2013). In this study, several important target genes participating in fruit ripening and senescence were found including Ethylene-responsive transcription factors, F-box proteins, MADS-box TFs, and MADS-box proteins.

### Network construction revealed the relationship of small RNAs and ethylene in tomato

**Figure 6:**
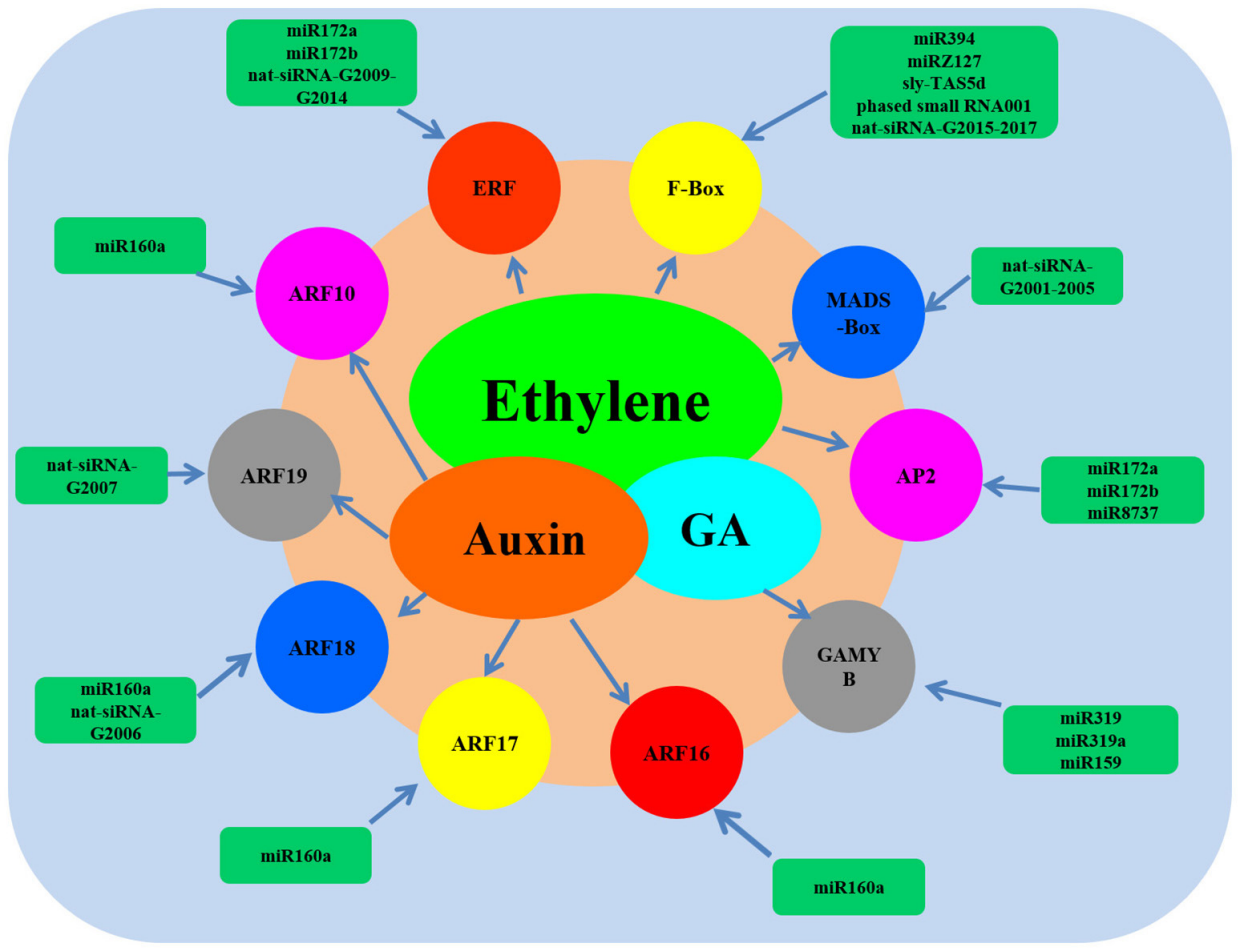
**Network model of the small RNAs and their target genes involved in ethylene**.

To illuminating the network between small RNAs and their target genes involved in ethylene, all the predicted target genes of miRNAs, ta-siRNAs, and nat-siRNAs were screened carefully and a regulatory model was set up (Figure 6). From the network model, it could clearly been seen that miR394, miRZ131, miR172, miR8737, miR319, miR159, miR160, and nat-siRNA-G2001 to nat-siRNA-G2017 as well as their target genes such as auxin response factors, ethylene-responsive transcription factors and GAMYB were involved in ethylene signal pathway. These results indicate that the network of miRNAs are quite complicated, and elucidation of the molecular mechanisms underlying the interplay between miRNA and their target genes involved in ethylene pathway requires further study.

## Conclusion

In summary, ethylene biosynthesis and signal transduction related miRNAs and siRNAs were identified in tomato fruit. These informations broaden the knowledge of the relationship between small RNAs and ethylene regulation. Additionally, many target genes of miRNAs were identified by bioinformatic prediction and degradome sequencing. The result showed that the target genes were involved in various functions and ethylene related targets were also discovered. In addition, a large amount of the target genes of nat-siRNAs were found and some of them were found to participate in ethylene regulation. A regulatory model which reveals the regulation relationship between the small RNAs and their targets was set up. Moreover, 41 putative novel miRNAs were identified and many of them were also involved in ethylene pathway. These findings lay the foundation for exploring the role of small RNAs in ethylene signaling pathway in the plant.

## Author contributions

JZ and LG designed the research; YW and JZ carried out the experiments; JZ, YW, QW, and ZJ analyzed the results; YW wrote the manuscript; JZ, BZ, and YL modified the manuscript; all authors have read and approved the manuscript for publication.

### Conflict of interest statement

The authors declare that the research was conducted in the absence of any commercial or financial relationships that could be construed as a potential conflict of interest.

## Supplementary material

Supplementary Figure S1 **The expressions of *LeERF1* wild (CK1, CK2), sense-*LeERF1* (F1, F2), and antisense-*LeERF1* (R1, R2) tomato fruit**.

Supplementary Figure S2 **The pipeline for the systematic identification of small RNAs in tomato**.

Supplementary Figure S3 **Expression profiles of the known and putative novel miRNAs in wild (CK1, CK2), overexpression sense-*LeERF1* (F1, F2), and antisense-*LeERF1* (R1, R2) tomato fruit**.

Supplementary Table S1 **Known miRNAs identified in tomato**.

Supplementary Table S2 **Less conserved miRNAs identified in tomato**.

Supplementary Table S3 **Putative novel miRNAs identified in tomato**.

Supplementary Table S4 **Phased small RNAs found in tomato**.

Supplementary Table S5 **Nat-siRNAs found in tomato**.

Supplementary Table S6 **Target genes of the miRNAs involved in ethylene by bioinformatic prediction**.

Supplementary Table S7 **Target genes of the miRNAs involved in ethylene by degradome sequencing**.

Supplementary Table S8 **Target genes of the nat-siRNAs involved in ethylene**.

## References

[B1] Addo-Quaye C. Eshoo T. W. Bartel D. P. Axtell M. J. (2008). Endogenous siRNA and miRNA targets identified by sequencing of the Arabidopsis degradome. Curr. Biol. 18, 758–762. 10.1016/j.cub.2008.04.042 18472421 PMC2583427

[B2] An F. M. Hsiao S. R. Chan M. T. (2011). Sequencing-based approaches reveal low ambient temperature- responsive and tissue-specific microRNAs in phalaenopsis orchid. PLoS ONE 6:e18937. 10.1371/journal.pone.0018937 21573107 PMC3089612

[B3] Anders S. Huber W. (2010). Differential expression analysis for sequence count data. Genome Biol. 11:R106. 10.1186/gb-2010-11-10-r106 20979621 PMC3218662

[B4] Axtell M. J. Snyder J. A. Bartell D. P. (2007). Common functions for diverse small RNAs of land plants. Plant Cell 19, 1750–1769. 10.1105/tpc.107.051706 17601824 PMC1955733

[B5] Baek D. Park H. C. Kima M. C. Yun D. J. (2013). The role of Arabidopsis MYB2 in miR399f-mediated phosphate-starvation response. Plant Signal. Behav. 8:e23488. 10.4161/psb.23488 23333957 PMC3676519

[B6] Candar-Cakir B. Arican E. Zhang B. H. (2016). Small RNA and degradome deep sequencing reveals drought-and tissue-specific micrornas and their important roles in drought-sensitive and drought-tolerant tomato genotypes. Plant Biotechnol. J. 14, 1727–1746. 10.1111/pbi.12533 26857916 PMC5067666

[B7] Cao X. Wu Z. Jiang F. Zhou R. Yang Z. (2014). Identification of chilling stress-responsive tomato microRNAs and their target genes by high-throughput sequencing and degradome analysis. BMC Genomics 15:1130. 10.1186/1471-2164-15-1130 25519760 PMC4377850

[B8] Carthew R. W. Sontheimer E. J. (2009). Origins and mechanisms of miRNAs and siRNAs. Cell 136, 642–655. 10.1016/j.cell.2009.01.035 19239886 PMC2675692

[B9] Chen H. M. Li Y. H. Wu S. H. (2007). Bioinformatic prediction and experimental validation of a microRNA-directed tandem trans-acting siRNA cascade in Arabidopsis. Proc. Natl. Acad. Sci. U.S.A. 104, 3318–3323. 10.1073/pnas.0611119104 17360645 PMC1805617

[B10] Chen X. (2009). Small RNAs and their roles in plant development. Annu. Rev. Cell Dev. Biol. 25, 21–44. 10.1146/annurev.cellbio.042308.113417 19575669 PMC5135726

[B11] Cheng H. Y. Wang Y. Tao X. Fan Y. F. Dai Y. Yang H.. (2016). Genomic profiling of exogenous abscisic acid-responsive microRNAs in tomato (*Solanum lycopersicum*). BMC Genomics 17:423. 10.1186/s12864-016-2591-8 27260799 PMC4891822

[B12] Chiou T. J. Aung K. Lin S. I. Wu C. C. Chiang S. F. Su C. L. (2006). Regulation of phosphate homeostasis by microRNA in Arabidopsis. Plant Cell 18, 412–421. 10.1105/tpc.105.038943 16387831 PMC1356548

[B13] Couzigou J. M. Combier J. P. (2016). Plant microRNAs: key regulators of root architecture and biotic interactions. New Phytol. 212, 22–35. 10.1111/nph.14058 27292927

[B14] Dalmay T. (2010). Short RNAs in tomato. J. Integr. Plant Biol. 52, 388–392. 10.1111/j.1744-7909.2009.00871.x 20377700

[B15] Fahlgren N. Howell M. D. Kasschau K. D. Chapman E. J. Sullivan C. M. Cumbie J. S.. (2007). High-throughput sequencing of Arabidopsis microRNAs: evidence for frequent birth and death of miRNA genes. PLoS ONE 2:e219. 10.1371/journal.pone.0000219 17299599 PMC1790633

[B16] Fujii H. Chiou T. J. Lin S. I. Aung K. Zhu J. K. (2005). A miRNA involved in phosphate-starvation response in Arabidopsis. Curr. Biol. 15, 2038–2043. 10.1016/j.cub.2005.10.016 16303564

[B17] Gao C. Ju Z. Cao D. Zhai B. Qin G. Zhu H.. (2015). MicroRNA profiling analysis throughout tomato fruit development and ripening reveals potential regulatory role of RIN on microRNAs accumulation. Plant Biotechnol. J. 13, 370–382. 10.1111/pbi.12297 25516062

[B18] Giovannoni J. J. (2004). Genetic regulation of fruit development and ripening. Plant Cell 16, S170–S180. 10.1105/tpc.019158 15010516 PMC2643394

[B19] Jain M. Chevala V. V. Garg R. (2014). Genome-wide discovery and differential regulation of conserved and novel microRNAs in chickpea via deep sequencing. J. Exp. Bot. 65, 5945–5958. 10.1093/jxb/eru333 25151616 PMC4203128

[B20] Jin W. Li N. Zhang B. Wu F. Li W. Guo A.. (2008). Identification and verification of microRNA in wheat (*Triticum aestivum*). J. Plant Res. 121, 351–355. 10.1007/s10265-007-0139-3 18357413

[B21] Jones-Rhoades M. W. Bartel D. P. Bartel B. (2006). MicroRNAs and their regulatory roles in plants. Annu. Rev. Plant Biol. 57, 19–53. 10.1146/annurev.arplant.57.032905.105218 16669754

[B22] Kanehisa M. Araki M. Goto S. Hattori M. Hirakawa M. Itoh M.. (2008). KEGG for linking genomes to life and the environment. Nucleic Acids Res. 36, D480–D484. 10.1093/nar/gkm882 18077471 PMC2238879

[B23] Li F. Orban R. Baker B. (2012). SoMART, a web server for plant miRNA, tasiRNA and target gene analysis. Plant J. 70, 891–901. 10.1111/j.1365-313X.2012.04922.x 22268718

[B24] Li Y. Zhu B. Xu W. Zhu H. Chen A. Xie Y.. (2007). *LeERF1* positively modulated ethylene triple response on etiolated seedling, plant development and fruit ripening and softening in tomato. Plant Cell Rep. 26, 1999–2008. 10.1007/s00299-007-0394-8 17639404

[B25] Li Y. F. Zheng Y. Addo-Quaye C. Zhang L. Saini A. Jagadeeswaran G.. (2010). Transcriptome-wide identification of microRNA targets in rice. Plant J. 62, 742–759. 10.1111/j.1365-313X.2010.04187.x 20202174

[B26] Li Z. H. Peng J. Y. Wen X. Guo H. W. (2013). Ethylene-insensitive3 is a senescence-associated gene that accelerates age-dependent leaf senescence by directly repressing miR164 transcription in Arabidopsis. Plant Cell 25, 3311–3328. 10.1105/tpc.113.113340 24064769 PMC3809534

[B27] Liu B. Li P. Li X. Liu C. Cao S. Chu C.. (2005). Loss of function of OsDCL1 affects microRNA accumulation and causes developmental defects in rice. Plant Physiol. 139, 296–305. 10.1104/pp.105.063420 16126864 PMC1203379

[B28] Liu H. H. Tian X. Li Y. J. Wu C. A. Zheng C. C. (2008). Microarray-based analysis of stress-regulated microRNAs in *Arabidopsis thaliana*. RNA 14, 836–843. 10.1261/rna.895308 18356539 PMC2327369

[B29] Lu C. W. Shao Y. Li L. Chen A. J. Xu W. Q. Wu K. J. (2011). Overexpression of *SlERF1* tomato gene encoding an ERF-type transcription activator enhances salt tolerance. Rus. J. Plant Physiol. 58, 118–125. 10.1134/S1021443711010092

[B30] Lu S. Sun Y. H. Chiang V. L. (2008). Stress-responsive microRNAs in Populus. Plant J. 55, 131–151. 10.1111/j.1365-313X.2008.03497.x 18363789

[B31] Mao X. Cai T. Olyarchuk J. Wei L. (2005). Automated genome annotation and pathway identification using the KEGG Orthology (KO) as a controlled vocabulary. Bioinformatics 21, 3787–3793. 10.1093/bioinformatics/bti430 15817693

[B32] Mohorianu I. Schwach F. Jing R. Lopez-Gomollon S. Moxon S. Szittya G.. (2011). Profiling of short RNAs during fleshy fruit development reveals stage-specific sRNAome expression patterns. Plant J. 67, 232–246. 10.1111/j.1365-313X.2011.04586.x 21443685

[B33] Morin R. D. Aksay G. Dolgosheina E. Ebhardt H. A. Magrini V. Mardis E. R.. (2008). Comparative analysis of the small RNA transcriptomes of Pinus contorta and *Oryza sativa*. Genome Res. 18, 571–584. 10.1101/gr.6897308 18323537 PMC2279245

[B34] Moxon S. Jing R. Szittya G. Schwach F. Rusholme Pilcher R. L. Moulton V.. (2008). Deep sequencing of tomato short RNAs identifies microRNAs targeting genes involved in fruit ripening. Genome Res. 18, 1602–1609. 10.1101/gr.080127.108 18653800 PMC2556272

[B35] Osorio S. Alba R. Damasceno C. M. Lopez-Casado G. Lohse M. Zanorc M. I.. (2011). Systems biology of tomato fruit development: combined transcript, protein and metabolite analysis of tomato transcription factor (nor, rin) and ethylene receptor (Nr) mutants reveals novel regulatory interactions. Plant Physiol. 157, 405–425. 10.1104/pp.111.175463 21795583 PMC3165888

[B36] Palatnik J. F. Allen E. Wu X. Schommer C. Schwab R. Carrington J. C.. (2003). Control of leaf morphogenesis by microRNAs. Nature 425, 257–263. 10.1038/nature01958 12931144

[B37] Pan X. Q. Fu D. Q. Zhu B. Z. Lu C. W. Luo Y. B. (2013). Overexpression of the ethylene response factor SlERF1 gene enhances resistance of tomato fruit to *Rhizopus nigricans*. Postharvest Biol. Technol. 75, 28–36. 10.1016/j.postharvbio.2012.07.008

[B38] Park J. M. Park C. J. Lee S. B. Ham B. K. Shin R. Paek K. H. (2001). Overexpression of the tobacco Tsi1 gene encoding an EREBP/AP2-type transcription factor enhances resistance against pathogen attack and osmotic stress in tobacco. Plant Cell 13, 1035–1046. 10.1105/tpc.13.5.1035 11340180 PMC135557

[B39] Pashkovskiy P. P. Ryazansky S. S. (2013). Biogenesis, evolution, and functions of plant microRNAs. Biochemistry 78, 627–637. 10.1134/s0006297913060084 23980889

[B40] Pirrello J. Jaimes-Miranda F. Sanchez-Ballesta M. T. Tournier B. Khalil-Ahmad Q. Regad F.. (2006). Sl-ERF2 a tomato ethylene response factor involved in ethylene response and seed germination. Plant Cell Physiol. 47, 1195–1205. 10.1093/pcp/pcj084 16857696

[B41] Qin J. Zhao J. Y. Zuo K. J. Cao Y. F. Ling H. Sun X. F. (2004). Isolation and characterization of an ERF-like gene from *Gossypium barbadense*. Plant Sci. 167, 1383–1389. 10.1016/j.plantsci.2004.07.012

[B42] Rajagopalan R. Vaucheret H. Trejo J. Bartel D. P. (2006). A diverse and evolutionarily fluid set of microRNAs in *Arabidopsis thaliana*. Genes Dev. 20, 3407–3425. 10.1101/gad.1476406 17182867 PMC1698448

[B43] Rhoades M. W. Reinhart B. J. Lim L. P. Burge C. B. Bartel B. Bartel D. P. (2002). Prediction of plant microRNA targets. Cell 110, 513–520. 10.1016/s0092-8674(02)00863-2 12202040

[B44] Rubio-Somoza I. Cuperus J. T. Weigel D. Carrington J. C. (2009). Regulation and functional specialization of small RNA-target nodes during plant development. Curr. Opin. Plant Biol. 12, 622–627. 10.1016/j.pbi.2009.07.003 19699140

[B45] Pilcher R. L. Moxon S. Pakseresht N. Moulton V. Manning K. Seymour G.. (2007). Identification of novel small RNAs in tomato (*Solanum lycopersicum*). Planta 226, 709–717. 10.1007/s00425-007-0518-y 17415587

[B46] Sanan-Mishra N. Kumar V. Sopory S. K. Mukherjee S. K. (2009). Cloning and validation of novel miRNA from basmati rice indicates cross talk between abiotic and biotic stresses. Mol. Genet. Genomics 282, 463–474. 10.1007/s00438-009-0478-y 20131478

[B48] Shivaprasad P. V. Chen H. M. Patel K. Bond D. M. Santos B. A. Baulcombe D. C. (2012). A microRNA superfamily regulates nucleotide binding site-leucine-rich repeats and other mRNAs. Plant Cell 24, 859–874. 10.1105/tpc.111.095380 22408077 PMC3336131

[B49] Sunkar R. Chinnusamy V. Zhu J. Zhu J. K. (2007). Small RNAs as big players in plant abiotic stress responses and nutrient deprivation. Trends Plant Sci. 12, 301–309. 10.1016/j.tplants.2007.05.001 17573231

[B50] Sunkar R. Girke T. Jain P. K. Zhu J. K. (2005). Cloning and characterization of microRNAs from rice(W). Plant Cell 17, 1397–1411. 10.1105/tpc.105.031682 15805478 PMC1091763

[B51] Talmor-Neiman M. Stav R. Frank W. Voss B. Arazi T. (2006). Novel micro-RNAs and intermediates of micro-RNA biogenesis from moss. Plant J. 47, 25–37. 10.1111/j.1365-313X.2006.02768.x 16824179

[B52] Thiebaut F. Rojas C. A. Grativol C. Motta M. R. Vieira T. Regulski M.. (2014). Genome-wide identification of microRNA and siRNA responsive to endophytic beneficial diazotrophic bacteria in maize. BMC Genomics 15:766. 10.1186/1471-2164-15-766 25194793 PMC4168055

[B53] Tomato Genome Consortium (2012). The tomato genome sequence provides insights into fleshy fruit evolution. Nature 485, 635–641. 10.1038/nature11119 22660326 PMC3378239

[B54] Wang H. Huang Z. Chen Q. Zhang Z. Zhang H. Wu Y. M.. (2004). Ectopic overexpression of tomato JERF3 in tobacco activates downstream gene expression and enhances salt tolerance. Plant Mol. Biol. 55, 183–192. 10.1007/s11103-004-0113-6 15604674

[B55] Wang X. Kong H. Ma H. (2009). F-box proteins regulate ethylene signaling and more. Genes Dev. 23, 391–396. 10.1101/gad.1781609 19240128

[B56] Wu G. Park M. Y. Conway S. R. Wang J. W. Weigel D. Scott Poethig R. (2009). The sequential action of miR156 and miR172 regulates developmental timing in Arabidopsis. Cell 138, 750–759. 10.1016/j.cell.2009.06.031 19703400 PMC2732587

[B57] Wu K. Q. Tian L. N. Hollingworth J. Brown D. C. W. Miki B. (2002). Functional analysis of tomato Pti4 in Arabidopsis. Plant Physiol. 128, 30–37. 10.1104/pp.010696 11788750 PMC148941

[B58] Wu P. Wu Y. Liu C. C. Liu L. W. Ma F. F. Wu X. Y.. (2016). Identification of Arbuscular Mycorrhiza (AM)-Responsive microRNAs in Tomato. Front. Plant Sci. 7:429. 10.3389/fpls.2016.00429 27066061 PMC4814767

[B59] Xie F. Jones D. C. Wang Q. Sun R. Zhang B. (2015). Small RNA sequencing identifies miRNA roles in ovule and fibre development. Plant Biotechnol. J. 13, 355–369. 10.1111/pbi.12296 25572837

[B60] Xie Z. Allen E. Fahlgren N. Calamar A. Givan S. A. Carrington J. C. (2005). Expression of Arabidopsis miRNA genes. Plant Physiol. 138, 2145–2154. 10.1104/pp.105.062943 16040653 PMC1183402

[B61] Yant L. Mathieu J. Dinh T. T. Ott F. Lanz C. Wollmann H.. (2010). Orchestration of the floral transition and floral development in Arabidopsis by the bifunctional transcription factor APETALA2. Plant Cell 22, 2156–2170. 10.1105/tpc.110.075606 20675573 PMC2929098

[B62] Yoshikawa M. Peragine A. Park M. Y. Poethig R. S. (2005). A pathway for the biogenesis of trans-acting siRNAs in Arabidopsis. Genes Dev. 19, 2164–2175. 10.1101/gad.1352605 16131612 PMC1221887

[B63] Young M. D. Wakefield M. J. Smyth G. K. Oshlack A. (2010). Gene ontology analysis for RNA-seq: accounting for selection bias. Genome Biol. 11:r14. 10.1186/gb-2010-11-2-r14 20132535 PMC2872874

[B64] Zhai J. X. Jeong D. H. De Paoli E. Park S. Rosen B. D. Li Y.. (2011). MicroRNAs as master regulators of the plant NB-LRR defense gene family via the production of phased, trans-acting siRNAs. Genes Dev. 25, 2540–2553. 10.1101/gad.177527.111 22156213 PMC3243063

[B65] Zhang J. Y. Broeckling C. D. Sumner L. W. Wang Z. Y. (2007). Heterologous expression of two *Medicago truncatula* putative ERF transcription factor genes, WXP1 and WXP2, in Arabidopsis led to increased leaf wax accumulation and improved drought tolerance, but differential response in freezing tolerance. Plant Mol. Biol. 64, 265–278. 10.1007/s11103-007-9150-2 17347795

[B66] Zhang R. X. Marshall D. Bryan G. J. Hornyik C. (2013). Identification and characterization of miRNA transcriptome in potato by high-throughput sequencing. PLoS ONE 8:e57233. 10.1371/journal.pone.0057233 23437348 PMC3578796

[B67] Zhang X. Zou Z. Zhang J. Zhang Y. Han Q. Hu T.. (2011). Over-expression of sly-miR156a in tomato results in multiple vegetative and reproductive trait alterations and partial phenocopy of the sft mutant. FEBS Lett. 585, 435–439. 10.1016/j.febslet.2010.12.036 21187095

[B68] Zhou X. F. Sunkar R. Jin H. Zhu J. K. Zhang W. X. (2009). Genome-wide identification and analysis of small RNAs originated from natural antisense transcripts in *Oryza sativa*. Genome Res. 19, 70–78. 10.1101/gr.084806.108 18971307 PMC2612963

[B69] Zuo J. Fu D. Zhu Y. Qu G. Tian H. Zhai B.. (2013). SRNAome parsing yields insights into tomato fruit ripening control. Physiol. Plant. 149, 540–553. 10.1111/ppl.12055 23550530

[B70] Zuo J. Zhu B. Fu D. Zhu Y. Ma Y. Chi L.. (2012). Sculpting the maturation, softening and ethylene pathway: the influences of microRNAs on tomato fruits. BMC Genomics 13:7. 10.1186/1471-2164-13-7 22230737 PMC3266637

[B71] Zuo J. H. Wang Q. Han C. Ju Z. Cao D. Zhu B.. (2016). SRNAome and degradome sequencing analysis reveals specific regulation of sRNA in response to chilling injury in tomato fruit. Physiol Plant. [Epub ahead of print]. 10.1111/ppl.12509 27595790

