# Supplementary figures and images for: Parsing the Regulatory Network between Small RNAs and Target Genes in Ethylene Pathway in Tomato
Source: Front Plant Sci. 2017 Apr 11;8:527. doi: 10.3389/fpls.2017.00527 (PMC5387102; doi:10.3389/fpls.2017.00527)

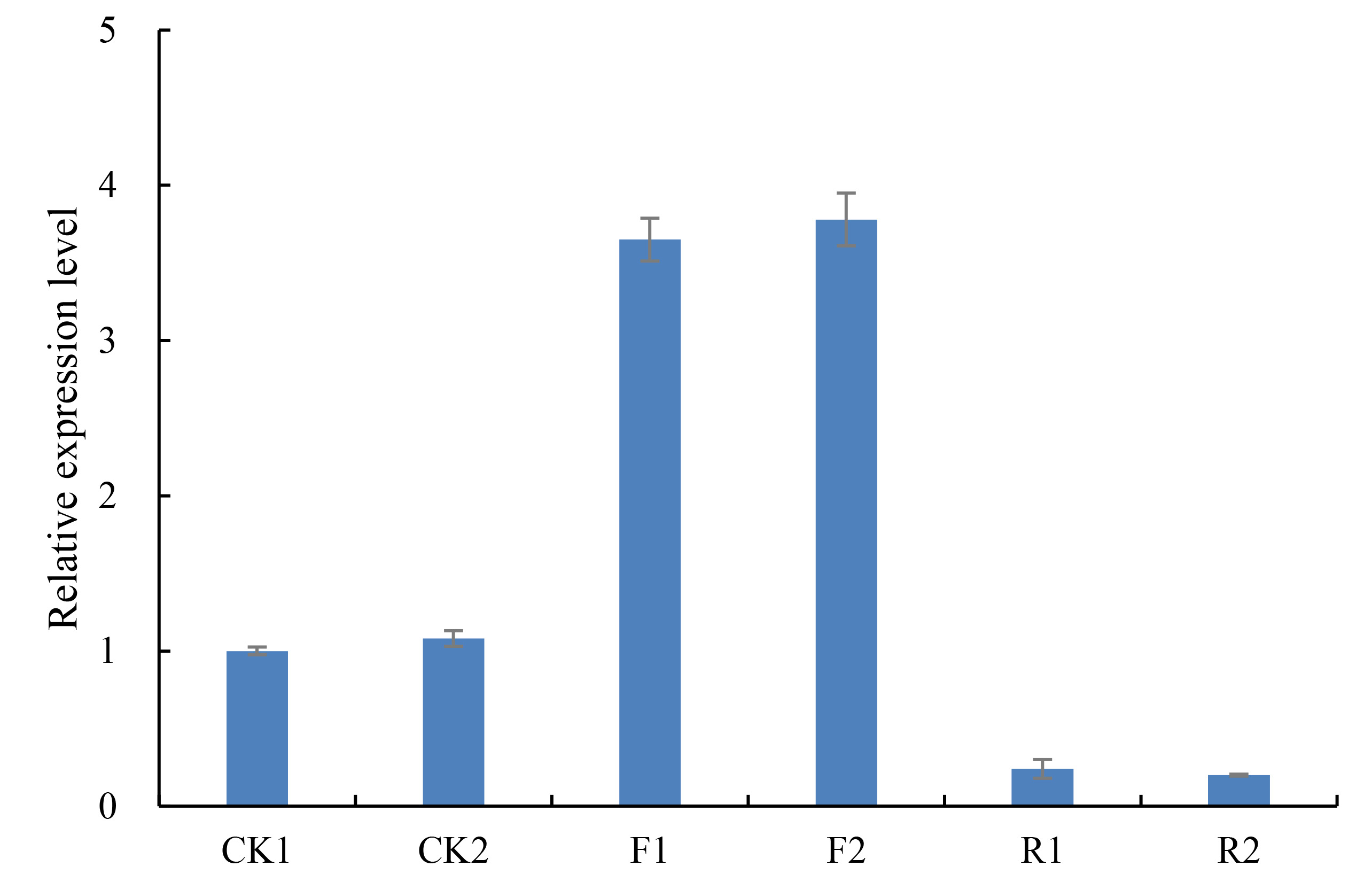

Supplement: Supplementary Figure S1 — The expressions of LeERF1 wild (CK1, CK2), sense-LeERF1 (F1, F2), and antisense-LeERF1 (R1, R2) tomato fruit. [file Image1.JPEG]

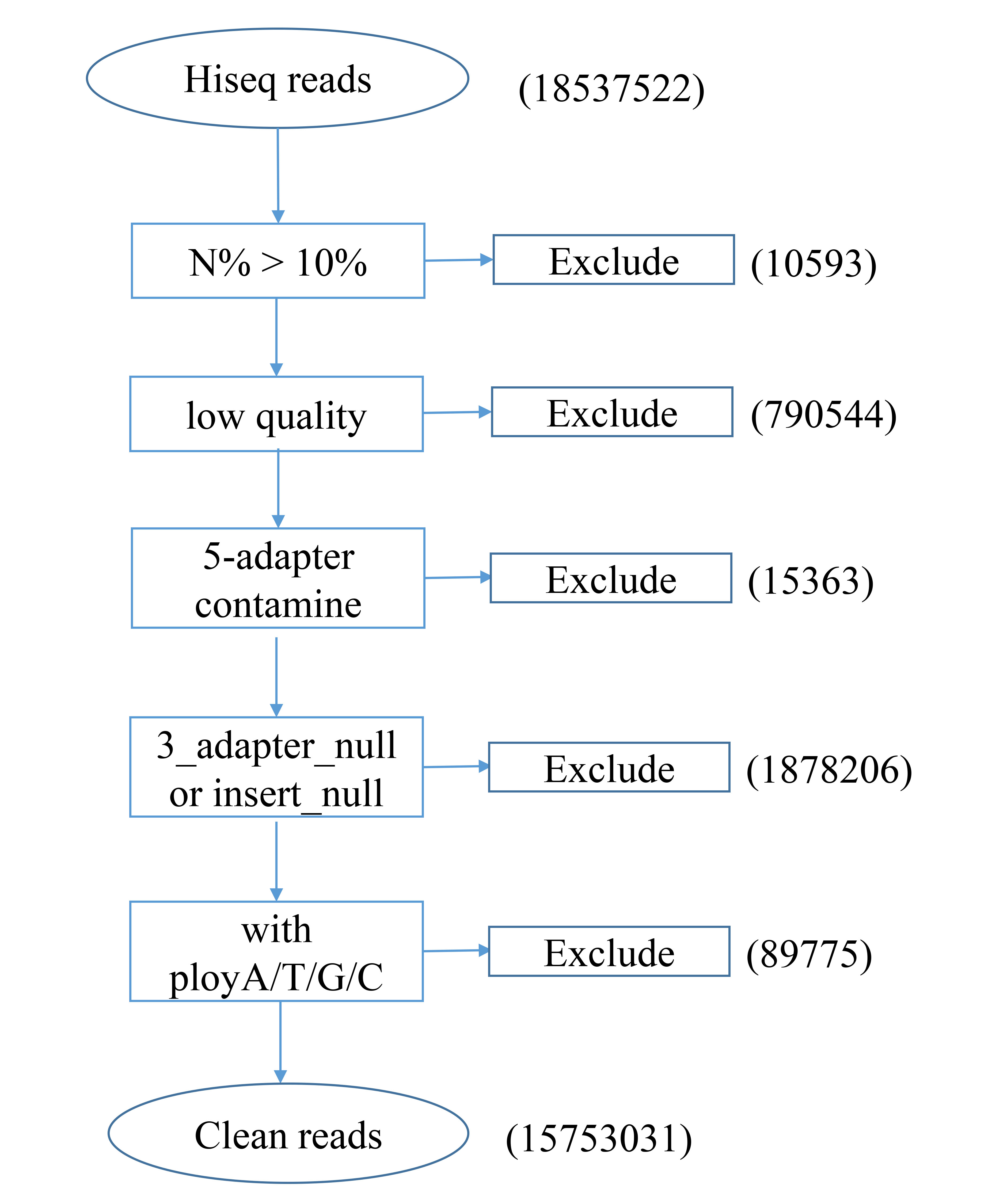

Supplement: Supplementary Figure S2 — The pipeline for the systematic identification of small RNAs in tomato. [file Image2.JPEG]

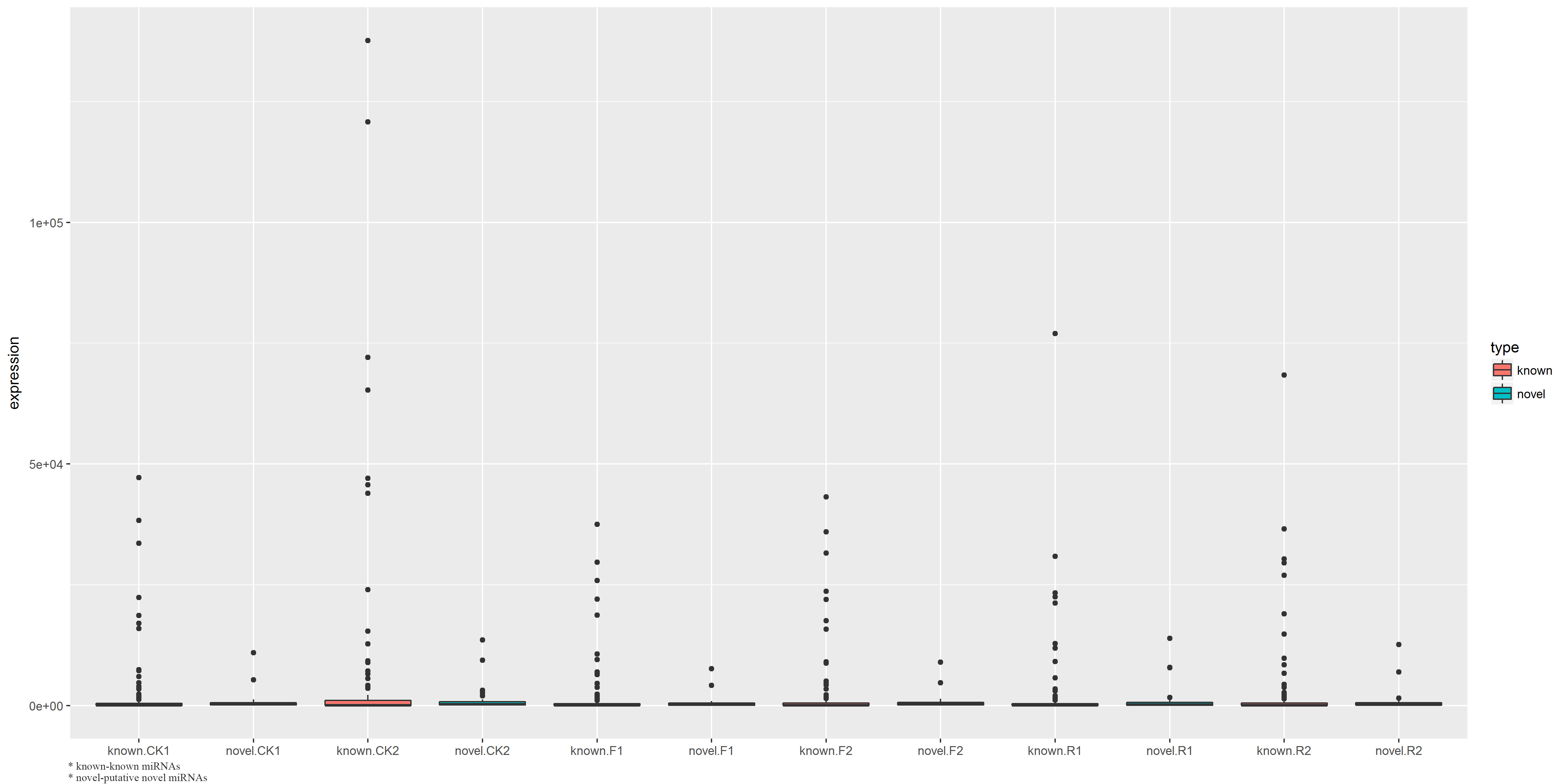

Supplement: Supplementary Figure S3 — Expression profiles of the known and putative novel miRNAs in wild (CK1, CK2), overexpression sense-LeERF1 (F1, F2), and antisense-LeERF1 (R1, R2) tomato fruit. [file Image3.JPEG]
